# Supplementary material for: Identification and functional analysis of non-coding regulatory small RNA FenSr3 in Bacillus amyloliquefaciens LPB-18
Source: PeerJ. 2023 May 15;11:e15236. doi: 10.7717/peerj.15236 (PMC10194069; doi:10.7717/peerj.15236)
Supplement: Supplemental Information 4 [file peerj-11-15236-s004.zip › KO/CK-vs-T1_map/map04218.html]

KEGG PATHWAY: Cellular senescence - Reference pathway


|  |  |
| --- | --- |
| **Cellular senescence - Reference pathway** |  |

[
Pathway menu
| Organism menu
| Pathway entry
| Show description
| User data mapping
]

Cellular senescence is a state of irreversible cellular arrest and can be triggered by a number of factors, such as telomere shortening, oncogene activation, irradiation, DNA damage and oxidative stress. It is characterized by enlarged flattened morphology, senescence-associated beta-galactosidase (SA-b-gal) activity, secretion of inflammatory cytokines, growth factors and matrix metalloproteinases, as part of the senescence-associated secretory phenotype (SASP). Cellular senescence is functionally associated with many biological processes including aging, tumor suppression, placental biology, embryonic development, and wound healing.

|  |  |  |
| --- | --- | --- |
| Reference pathway | 184% 150% 122% 100% 82% 67% 55% | 图片下载 |
